# Placebo effects on nausea and motion sickness are resistant to experimentally-induced stress

**DOI:** 10.1038/s41598-023-36296-w

**Published:** 2023-06-19

**Authors:** Carmen Jacob, Elisabeth Olliges, Anja Haile, Verena Hoffmann, Benjamin Jacobi, Leander Steinkopf, Marina Lanz, Marc Wittmann, Matthias H. Tschöp, Karin Meissner

**Affiliations:** 1 Institute of Medical Psychology, Medical Faculty, LMU Munich, 80336 Munich, Germany; 2 Wessex Neurological Centre, University Hospital Southampton and Clinical Neurosciences, Clinical and Experimental Sciences, Faculty of Medicine, University of Southampton, Southampton General Hospital, Southampton, SO16 6YD UK; 3 Department of Psychosomatic Medicine and Psychotherapy, Klinik Barmelweid AG, 5017 Barmelweid, Switzerland; 4 Department of Psychiatry, University of Connecticut Health Center, H1010, Farmington, CT 06030-1410 USA; 5 Institute for Frontier Areas of Psychology and Mental Health, 79098 Freiburg, Germany; 6 Institute for Diabetes and Obesity, Helmholtz Diabetes Center, Helmholtz Zentrum München, 85764 Neuherberg, Germany; 7 Division of Metabolic Diseases, Department of Medicine, Technische Universität München, 81675 Munich, Germany; 8 Division of Health Promotion, Coburg University of Applied Sciences and Arts, Friedrich-Streib-Str. 2, 96450 Coburg, Germany

**Keywords:** Social neuroscience, Gastrointestinal system, Gastrointestinal diseases

## Abstract

Nausea often occurs in stressful situations, such as chemotherapy or surgery. Clinically relevant placebo effects in nausea have been demonstrated, but it remains unclear whether stress has an impact on these effects. The aim of this experimental study was to investigate the interplay between acute stress and placebo effects in nausea. 80 healthy female volunteers susceptible to motion sickness were randomly assigned to either the Maastricht Acute Stress Test or a non-stress control condition, and to either placebo treatment or no treatment. Nausea was induced by a virtual vection drum and behavioral, psychophysiological as well as humoral parameters were repeatedly assessed. Manipulation checks confirmed increased cortisol levels and negative emotions in the stressed groups. In the non-stressed groups, the placebo intervention improved nausea, symptoms of motion sickness, and gastric myoelectrical activity (normo-to-tachy (NTT) ratio). In the stressed groups, the beneficial effects of the placebo intervention on nausea and motion sickness remained unchanged, whereas no improvement of the gastric NTT ratio was observed. Results suggest that placebo effects on symptoms of nausea and motion sickness are resistant to experimentally-induced stress. Stress most likely interfered with the validity of the gastric NTT ratio to measure nausea and thus the gastric placebo effect.

## Introduction

Placebo effects occur not only after placebo interventions, but also contribute to the overall effect of almost any treatment^1^. A central goal of placebo research is therefore to better understand how contextual factors influence placebo effects. A little explored contextual factor in this regard is stress, which is ubiquitous in clinical settings.

According to the biopsychosocial model of arousal, stress integrates biological, psychological, and social factors^2^. Biological factors refer to the physiological responses during stress, such as the release of the stress hormones cortisol and adrenaline, which prepare the body to deal with the perceived threat. Psychological factors encompass emotional responses such as fear and anxiety, as well as cognitive appraisal of the stressful situation, which can further modify the stress response. Social factors refer to the social and environmental influences on the stress response such as interactions with others and social support from family and friends, which can buffer the effects of stress. Stress results from a dynamic interaction between these biological, psychological and social components^2^.

Placebo effects are likewise based on mutual interactions between biological, psychological and social factors. Research has shown, for example, that placebo interventions trigger physiological responses, such as the release of endogenous opioids, resulting in reduced pain perception^3^. Other studies have demonstrated placebo-induced changes in immune system functioning^4^ as well as cardiovascular and gastrointestinal responses^5^. Furthermore, psychological and cognitive dimensions play a central role in placebo effects. For example, if a person believes a sugar pill to be a powerful painkiller, the expectation of pain relief can lead to an actual reduction in pain intensity^6^. Social factors, including the patient's relationship with the healthcare provider and the level of social support, can further shape the psychological and biological placebo response^7^.

Given the complex interplay between biological, psychological and cognitive factors in both the stress response and the placebo effect, it is reasonable to suppose that both phenomena can interfere with each other. For example, findings from placebo research in the field of pain suggest that placebo interventions decrease experimental pain by reducing anticipatory stress and anxiety^8,9^. Furthermore, the presence of an additional stressor may further increase negative emotions, resulting in a smaller placebo effect on pain due to larger stress. Indeed, Lyby et al.^10^ induced acute fear in healthy volunteers by the anticipation of electrical shocks and demonstrated that the placebo effect on experimental pain was diminished by fear. However, a recent study by Roderigo et al.^11^ showed different results, as a stressful state induced by the Trier Social Stress Test enhanced placebo effects on urgency-to-defecate but did not affect visceral placebo hypoalgesia. The conflicting results may be due in part to different placebo effects and mechanisms for different symptoms and organ systems.^5,12^

In a series of studies, we recently investigated placebo effects in an experimental nausea paradigm and could show that a suitable placebo intervention combined with verbal suggestions significantly reduced nausea and motions sickness in healthy volunteers^13,14^. In addition, the placebo effect on nausea was associated with improved gastric activity in women as well as with meaningful changes of blood proteins^15^, indicating that the placebo effect in nausea can at least partially be objectively assessed. Our results were consistent with those of several studies demonstrating placebo effects on nausea and motion sickness in experimental and clinical settings^13,14,16,17^, although some studies have also shown negative or mixed results^18–20^. In patients, nausea often occurs in situations associated with high stress and apprehension per se, including medical interventions such as chemotherapy, radiotherapy, and surgical procedures^21^. Therefore, a better understanding of the interplay between acute stress and placebo effects in nausea could help to improve the management of this common, yet hard to treat symptom^22^.

In this study, we investigated for the first time the effects of negative emotions induced by acute experimental stress on placebo effects in nausea using an established placebo nausea paradigm^13,14^. Our aim was to induce significant stress, including the release of cortisol, as this may best reflect the intense stress of patients in a clinical setting. We elicited acute stress by the Maastricht Acute Stress Test (MAST), which is a validated stress protocol known to induce robust emotional and neuroendocrine responses; a validated control version of the test is also available^23^. Based on the original findings by Lyby et al. that acute fear reduced the effectiveness of placebo analgesia^10^, we hypothesized that a state of acute stress going along with negative emotions would diminish placebo effects on nausea and motion sickness, and, consequently, also the placebo-related improvement of the gastric correlate of nausea. We focused on female participants, as our previous placebo nausea study showed gastric placebo effects only in women^14,15^.

In addition to nausea, we explored for the first time how acute stress and the placebo intervention might influence time estimation in nausea. The rationale for including time estimation as an outcome was based on previous findings that time perception is modulated by acute stress and anxiety^24,25^ and by symptoms such as pain^26^.

## Results

### Participants

94 women participated in the study, of whom 12 women were assigned to real treatment (data not analysed), and two women dropped out on the testing day before randomization (1 withdrew consent, 1 developed circulatory problems during baseline measurement). Analyses were based on data from 80 participants assigned to placebo treatment (n = 41; 21 no stress, 20 stress) or no treatment (n = 39; 20 no stress, 19 stress). Participants were on average 24.3 years old (SD 3.2) and had received 17.6 years of formal education (SD 3.1). 95% were German native speakers; all participants were fluent in German. The majority of participants were Caucasian; 3 participants were of Asian descent. About two thirds (63%) of the participants used hormonal contraceptives. Sample characteristics at baseline are summarized in Table 1.

**Table 1:** Sample characteristics at baseline.

	No stress, no treatment (n = 20)	No stress, placebo treatment (n = 21)	Stress, no treatment (n = 19)	Stress, placebo treatment (n = 20)	*p*-value^a^
Age (years), *mean (SD)*	24.25 (2.95)	24.86 (3.45)	23.5 (3.9)	24.39 (2.48)	0.632
Years of formal education, *mean (SD)*	17.95 (3)	17.98 (3.49)	17.11 (3.82)	17.29 (2.11)	0.766
Body Mass Index (kg/m^2^), *mean (SD)*	21.09 (1.89)	20.78 (1.47)	21.29 (2.39)	21.19 (2.15)	0.858
Use of hormonal contraception, *n (%)*	10 (50)	14 (67)	13 (68)	13 (65)	0.609^b^
Day in menstrual cycle^c^, *mean (SD)*	17.89 (9.26)	16.53 (8.8)	14.79 (7.84)	15.42 (8.02)	0.696
Blood sugar (mg/dl), *mean (SD)*	94.78 (10.52)	88.2 (24.72)	94.59 (16.92)	89.12 (14.69)	0.552
MSSQ, mean (SD)	136.71 (31.82)	127.4 (40.24)	143.64 (44.7)	126.96 (36.47)	0.493
Nausea during screening session (NRS 0–10), *mean (SD)*	6.45 (1.08)	6.19 (1.19)	6.29 (0.98)	6.44 (1.17)	0.854
HADS anxiety*, mean (SD)*	4.3 (2.27)	3.38 (2.22)	3.95 (1.84)	3.28 (2.63)	0.448
HADS depression, *mean (SD)*	1.95 (2.11)	1.81 (1.78)	1.32 (1.49)	1.11 (1.45)	0.397
STAI-State, *mean (SD)*	34.6 (6.21)	33.24 (6.46)	32.63 (5.8)	32.7 (7.26)	0.757
Expectation ratings (NRS 0–10), *mean (SD)*	7.10 (1.25)	7.62 (1.56)	7.32 (0.95)	7.45 (1)	0.582

^a^ANOVA if not otherwise indicated; ^b^Chi-square test; ^c^for participants not on hormonal birth control (“0” = day 1 of menstruation). *SD*, Standard deviation; *MMSQ*, Motion Sickness Susceptibility Questionnaire; *NRS*, Numeric rating scale; *HADS*; Hospital Anxiety and Depression Scale; *STAI*, State-Trait-Anxiety Inventory.

### Nausea-related measures

After the placebo intervention, the expected severity of nausea, corrected for baseline levels, was significantly lower in the placebo groups compared with the control groups (analysis of covariance (ANCOVA) main effect of *treatment*, *F*(1,75) = 10.44, *p* = 0.002, *η*_*p*_^*2*^ = 0.12; Table 2), thus confirming the induction of positive treatment expectations by the placebo intervention. Stress had no effect on nausea expectation (main effect of *stress*, *F*(1,75) = 0.05, *p* = 0.826, *η*_*p*_^*2*^ = 0) and also did not interact with treatment (*stress x treatment* interaction, *F*(1,75) = 0.32, *p* = 0.575, *η*_*p*_^*2*^ = 0).

**Table 2:** Outcome variables in the four experimental groups (estimated marginal means (SE) from analyses of covariance, with baseline values included as covariate).

	No stress, no treatment (n = 19)	No stress, placebo treatment (n = 19)	Stress, no treatment (n = 19)	Stress, placebo treatment (n = 20)
Expectation ratings (NRS 0–10)	7.32 (0.17)	6.86 (0.17)	7.37 (0.17)	6.73 (0.17)
Mean nausea ratings (NRS 0–10)	5 (0.43)	2.76 (0.42)	5.7 (0.45)	2.66 (0.43)
SSMS scores	9.45 (0.74)	6.4 (0.73)	8.4 (0.76)	5.44 (0.74)
Gastric NTT ratio	0.78 (0.11)	1.09 (0.1)	1.08 (0.11)	0.93 (0.11)
Time estimation (VAS 0–10)	3.54 (0.53)	5.71 (0.51)	5.53 (0.53)	4.69 (0.51)

*SE*, standard error; *NRS*, numeric rating scale; *SSMS*, Subjective symptoms of motion sickness questionnaire; *NTT*, normo-to-tachy; *VAS*, visual analogue scale.

**Figure 1:**
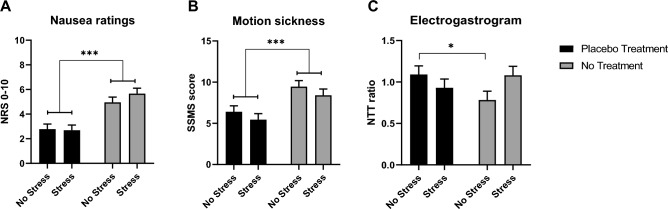
Mean effects (*SE*) of placebo treatment on (**A**) nausea ratinfigs, (**B**) motion sickness (SSMS), and (**C**) the gastric NTT-ratio in the stressed and the non-stressed groups. *Note:* *p < 0.05; ***p < 0.001 (Bonferroni-corrected post hoc tests). *Abbreviations*: *SE*, standard error; *SSMS*, Symptoms of Motion Sickness; *NTT,* normo-to-tachy.

The average intensity of nausea, corrected for baseline levels, was significantly lower in placebo-treated participants than in untreated participants (ANCOVA main effect of *treatment*, *F*(1,75) = 35.73, *p* < 0.001, *η*_*p*_^*2*^ = 0.32; Table 2, Fig. 1A). Stress had no effect on nausea (main effect of *stress, F*(1,75) = 0.59, *p* = 0.445, *η*_*p*_^*2*^ = 0.01) and did not interact with the placebo effect on nausea (*stress x treatment* interaction, *F*(1,75) = 0.967, *p* = 0.329, *η*_*p*_^*2*^ = 0.01).

Placebo treatment significantly reduced scores in the Subjective Symptoms of Motion Sickness questionnaire (SSMS, corrected for baseline levels; ANCOVA main effect of treatment, *F*(1,75) = 16.42, *p* < 0.001, *η*_*p*_^*2*^ = 0.18; Table 2, Fig. 1B). Stress induction had no effect on motion sickness (main effect of *stress F*(1,75) = 1.81, *p* = 0.183, *η*_*p*_^*2*^ = 0.02) and did not interact with the placebo effect on SSMS scores (*stress x treatment* interaction, *F*(1,75) = 0.003, *p* = 0.954, *η*_*p*_^*2*^ = 0).

The ANCOVA for the gastric normo-to-tachy (NTT) ratio during the target period, corrected for baseline levels, showed a significant interaction between *stress* and *treatment* (*F*(1,75) = 4.68, *p* = 0.034, *η*_*p*_^*2*^ = 0.06; Table 2, Fig. 1C). Bonferroni-corrected post hoc tests indicated a placebo effect in the non-stressed groups (*p* = 0.041), which was not observed in stressed groups (*p* = 0.323). Furthermore, stress tended to increase the gastric NTT ratio in untreated individuals (*p* = 0.056), but not in placebo-treated individuals (*p* = 0.287). Neither stress nor treatment had an independent effect on the gastric NTT ratio (main effect of *stress*, *F*(1,75) = 0.41, *p* = 0.525, *η*_*p*_^*2*^ = 0.01; main effect of *treatment*, *F*(1,75) = 0.55, *p* = 0.462, *η*_*p*_^*2*^ = 0.01).

### Time estimation

The two-way analysis of variance (ANOVA) for time estimation after nausea revealed a significant *stress x treatment* interaction (*F*(1,74) = 8.35, *p* = 0.005, *η*_*p*_^*2*^ = 0.1; Table 2). Bonferroni-corrected post hoc tests indicated a perception of faster passage of time after placebo administration in the non-stressed groups (*p* = 0.004) but not in the stressed groups (*p* = 0.258). At the same time, stress accelerated the subjective passage of time in the untreated groups (*p* = 0.009), but not in the placebo-treated groups (*p* = 0.165). Neither stress nor treatment had an independent effect on time estimation (main effect of *stress*, *F*(1,74) = 0.87, *p* = 0.354, *η*_*p*_^*2*^ = 0.01; main effect of *treatment*, *F*(1,74) = 1.63, *p* = 0.205, *η*_*p*_^*2*^ = 0.02).

### Salivary cortisol and negative emotions

**Figure 2:**
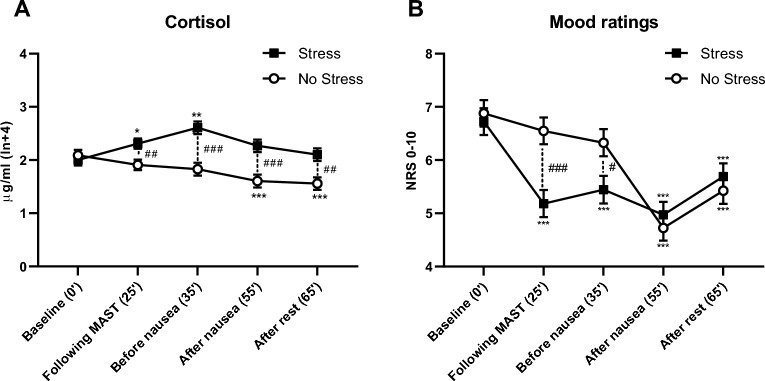
Mean salivary cortisol levels (**A**) and mood ratings (**B**) at baseline, following the Maastricht Acute Stress Test (MAST), before nausea induction, after nausea induction, and at the end of the experiment. *Note:* **p < 0.01; ***p < 0.001 (Bonferroni-corrected post hoc tests for within-group comparisons); ^#^*p* < 0.05; ^##^*p* < 0.01; ^###^*p* < 0.001, (Bonferroni-corrected post hoc tests for between-group comparisons);. Error bars indicate standard error. Abbreviations: *NRS*: Numeric rating scale.

The mixed-design ANOVA for cortisol indicated a significant *stress* x *time* interaction (*F*(2.23, 162.39) = 17.46, *p* < 0.001, *η*_*p*_^*2*^ = 0.19), which was due to significantly higher cortisol levels in the stressed groups than in non-stressed groups at all assessments after the MAST (Bonferroni-corrected *p*-values: *p* < 0.001, *p* < 0.001, *p* = 0.002, respectively; Table 3, Fig. 2A). In the stressed groups, cortisol levels were significantly higher after the MAST and before the onset of the vection stimulus compared with baseline values (Bonferroni-corrected *p*-values *p* = 0.002 and *p* = 0.001, respectively). In the non-stressed groups, cortisol levels declined during the experiment, with significantly lower levels after nausea and rest than during baseline (Bonferroni-corrected *p*-values < 0.001). The *treatment* x *time* interaction was not significant (*F*(2.23, 162.39) = 0.46, *p* = 0.652, *η*_*p*_^*2*^ = 0.01), nor was the threefold interaction *stress x time x treatment* (*F*(2.23, 162.39) = 0.49, *p* = 0.630, *η*_*p*_^*2*^ = 0.01).

**Table 3:** Stress variables (means, SE) during the experiment.

	Baseline	After MAST	Before nausea	After nausea	After rest
**Salivary cortisol [ln + 4 μg/ml]**, *mean (SE)*					
No stress, no treatment (n = 19)	2.08 (0.15)	1.95 (0.14)	1.96 (0.17)	1.69 (0.17)	1.63 (0.17)
No stress, placebo treatment (n = 19)	2.1 (0.15)	1.87 (0.14)	1.7 (0.17)	1.52 (0.17)	1.48 (0.17)
Stress, no treatment (n = 19)	2.08 (0.15)	2.39 (0.14)	2.67 (0.17)	2.38 (0.17)	2.2 (0.17)
Stress, placebo treatment (n = 20)	1.93 (0.14)	2.23 (0.14)	2.55 (0.17)	2.15 (0.17)	2 (0.16)
**Mood ratings (NRS 0–10)**, *mean (SE)*					
No stress, no treatment (n = 20)	6.25 (0.35)	5.8 (0.35)	5.35 (0.36)	3.8 (0.34)	4.65 (0.35)
No stress, placebo treatment (n = 20)	7.5 (0.35)	7.3 (0.35)	7.3 (0.36)	5.65 (0.34)	6.2 (0.35)
Stress, no treatment (n = 19)	6.95 (0.36)	5.32 (0.36)	5.79 (0.37)	4.9 (0.35)	5.63 (0.36)
Stress, placebo treatment (n = 20)	6.5 (0.35)	5.1 (0.35)	5.1 (0.36)	5.05 (0.34)	5.75 (0.35)
**State anxiety (STAI)**, *mean (SE)*					
No stress, no treatment (n = 20)	34.6 (1.45)	27.15 (2.18)	n/a	n/a	40.9 (1.76)
No stress, placebo treatment (n = 20)	33 (1.45)	32 (2.18)	n/a	n/a	35 (1.76)
Stress, no treatment (n = 19)	32 6 (1.49)	44.21 (2.24)	n/a	n/a	44.21 (2.24)
Stress, placebo treatment (n = 20)	32.7 (1.45)	45.4 (2.18)	n/a	n/a	35.9 (1.76)
**Inner-tension ratings (NRS 0–10)**, *mean (SE)*					
No stress, no treatment (n = 20)	1.40 (0.61)	1.65 (0.6)	1.53 (0.55)	5.50 (0.44)	3.30 (0.42)
No stress, placebo treatment (n = 20)	0.81 (0.15)	0.70 (0.25)	1.71 (0.47)	4.10 (0.43)	2.00 (0.46)
Stress, no treatment (n = 19)	0.79 (0.21)	2 (0.44)	1.21 (0.3)	5.32 (0.43)	2.53 (0.42)
Stress, placebo treatment (n = 20)	1.10 (0.32)	2.75 (0.37)	1.95 (0.37)	4.05 (0.63)	2.10 (0.45)

*SE*, standard error; *MAST*, Maastricht Acute Stress Test; *NRS*, numeric rating scale; *STAI*, State Trait Anxiety Inventory.

The mixed-design ANOVA for mood ratings indicated a significant *stress* x *time* interaction (*F*(3.49, 261.56) = 8.57, *p* < 0.001, *η*_*p*_^*2*^ = 0.1), which was due to significantly lower mood ratings in the stressed compared to the non-stressed groups after the MAST and before the onset of the vection stimulus (Bonferroni-corrected *p* < 0.001 and *p* = 0.018, respectively; Table 3, Fig. 2B). In the stressed groups, mood levels decreased from baseline to all follow-up measurements (all Bonferroni-corrected *p*-values < 0.001), while in the non-stressed groups, mood decreased significantly only from baseline to nausea and rest (Bonferroni-corrected *p*-values < 0.001). The *treatment* x *time* interaction was not significant (*F*(3.49, 261.56) = 0.86, *p* = 0.474, *η*_*p*_^*2*^ = 0.01), nor was the interaction *stress x time x treatment* (*F*(3.49, 261.56) = 0.88, *p* = 0.468, *η*_*p*_^*2*^ = 0.01).

The mixed-design ANOVA for state anxiety indicated a significant *stress x time* interaction (*F*(1.85,138.9) = 24.57, *p* < 0.001, *η*_*p*_^*2*^ = 0.25), which was due to higher state anxiety levels after the MAST in the stressed compared to the non-stressed participants (Bonferroni-corrected *p* < 0.001; Table 3). In the stressed participants, state anxiety increased from baseline to MAST (Bonferroni-corrected *p* < 0.001) and rest (Bonferroni-corrected *p* = 0.014). In the non-stressed participants, state anxiety increased from baseline to rest (Bonferroni-corrected *p* = 0.002). The *treatment* x *time* interaction was not significant (*F*(1.85,138.9) = 0.75, *p* = 0.467, *η*_*p*_^*2*^ = 0.01), nor was the threefold interaction *stress x time x treatment* (*F*(1.85,138.9) = 0.84, *p* = 0.426, *η*_*p*_^*2*^ = 0.01).

The mixed-design ANOVA for ratings of inner tension indicated a significant *stress x time* interaction (*F*(3.35, 247.77) = 3.32, *p* = 0.017, *η*_*p*_^*2*^ = 0.04), with significantly higher ratings of inner tension after the MAST in the stressed compared to the non-stressed groups (Bonferroni-corrected *p* = 0.01; Table 3). In the stressed groups, inner tension increased from baseline to MAST, nausea, and rest (Bonferroni-corrected *p*-values, *p* < 0.001, *p* < 0.001, *p* = 0.002, respectively). In the non-stressed groups, inner tension increased from baseline to nausea and rest (Bonferroni-corrected *p* < 0.001). In addition, a significant *treatment x time* interaction was observed (*F*(3.35, 247.77) = 3.77, *η*_*p*_^*2*^ = 0.05), with significantly lower tension ratings after nausea induction in placebo-treated participants compared with untreated ones (Bonferroni-corrected *p* = 0.009; Table 3). The threefold interaction *stress x time x treatment* was not significant (*F*(3.35, 247.77, *η*_*p*_^*2*^ = 0.02) = 1.09, *p* = 0.357).

### Explorative correlations

To better understand the relationships between the gastric NTT ratio and nausea, motion sickness, stress, negative emotions, and time estimation, explorative correlation analyses of parameter changes from baseline to nausea were performed for the stressed and the non-stressed groups (Table 4). In the non-stressed groups, the gastric NTT ratio correlated negatively with nausea (*r* = -0.35, *p* = 0.025), whereas in the stressed groups, a positive association between the gastric NTT ratio and ratings of inner tension emerged (*r* = 0.33, *p* = 0.042).

**Table 4:** Pearson’s correlational coefficients for relationships between changes from baseline to the target period (∆) for the gastric NTT ratio and nausea indices, negative emotions, cortisol, and time perception in the stressed and the non-stressed groups.

	No stress (n = 41)	Stress (n = 39)
∆ NTT ratio	1	1
∆ Nausea ratings (NRS 0–10)	− 0,35*	0,16
∆ SSMS scores	− 0,27	0,3
∆ Mood ratings (NRS 0–10)	0,31	0,02
∆ Inner-tension ratings (NRS 0–10)	− 0,31	0,33*
∆ Cortisol [ln + 4 μg/ml], *mean (SE)*	− 0,24	−0,03
∆ Time perception (VAS 0–10)	0,18	0,14

*NTT*, normo-to-tachy; *NRS*, numeric rating scale; *SSMS,* Subjective Symptoms of Motion Sickness; *VAS,* Visual analogue scale. *p < 0.05.

Significant values are in [bold].

## Discussion

This is the first study to investigate the interplay between acute stress and placebo effects in nausea. In line with previous studies^13,14,16^, the expectancy manipulation induced a strong placebo effect on the severity of nausea and motion sickness in the non-stressed groups. We also replicated our recent finding of a placebo effect on the gastric NTT-ratio in a female population in the absence of experimental stress^15^. The novel finding of the present study is that in the stressed groups, the placebo effect on nausea and motion sickness remained unaffected, while a placebo effect on the gastric NTT ratio was no longer observed.

Using a visceral pain model, Roderigo et al.^11^ recently demonstrated that acute stress had no impact on placebo analgesia, but enhanced the placebo effect on urgency-to-defecate. Our results partially confirm these results, as acute stress did not affect the placebo effect at the level of symptoms, i.e., nausea and motion sickness. Thus, placebo effects on symptom perception, evaluation and reporting appear to be robust against acute stress. However, our results suggest that gastric myoelectric activity responded differently to placebo treatment depending on whether acute stress was induced or not. Only in the non-stressed participants, and consistent with previous studies^27,28^, a *negative* association between inner-tension ratings and the gastric NTT ratio emerged, indicating that larger stress was associated with less normal gastric activity. In the stressed groups, a *positive* association between inner-tension ratings and the gastric NTT ratio was found, suggesting that larger stress was associated with increased normal gastric activity. Several studies indicate that laboratory stressors such as shock avoidance, speech preparation, hand grip, cold stress, audio stress, Stroop task, and arithmetic tasks decrease the spectral power in the normogastric frequency band and/or increase it in the tachygastric frequency band^29–32^, resulting in a reduced NTT ratio. Other studies, however, reported different results and concluded that the direction of changes to acute stress may depend on the fasting state of the participants: after a meal, when normal gastric motility is high, the inhibitory effects of stress on gastric myoelectrical activity are usually large and significant, while in the fasting state, when gastric motility is low, the effects of stress on gastric myoelectrical activity may be absent, or even go in the opposite direction^33,34^. Since our experiments were performed in the fasting state, stress may have induced a paradoxical increase of normal gastric motility, thereby interfering with the validity of the gastric NTT ratio as a measure of nausea.

The question of whether placebo-induced expectations can affect peripheral physiological systems remains controversial. Although autonomic changes can be reliably induced by placebo interventions^35,36^, it is unclear whether these changes form an intrinsic part of the neurobiological placebo response or represent autonomic correlates of symptoms such as pain and nausea, which are expected to decrease when symptoms improve^5^. Furthermore, in a clinical trial of asthma patients, a placebo inhaler was found to improve asthma symptoms while lung function did not change (FEV1)^37^, suggesting that beyond autonomic correlates, placebo interventions may have no effects on pathophysiological processes. With this in mind, it would be interesting to investigate additional, more sensitive physiological parameters to elucidate the nature of peripheral placebo responses. In a recent study, for example, we used proteomics analyses of peripheral blood samples to identify protein signatures associated with the placebo effect in nausea^15^. Among other changes, placebo effects were related to alterations in micro-inflammatory proteins and neuropeptides such as neurexin. Various biological pathways were found to be involved, including the acute-phase response as well as regulation of grooming behavior^15^. Further studies of this type could help to clarify the extent to which peripheral changes are part of the neurobiological placebo response or rather consequences of reduced stress and autonomic arousal.

In an exploratory approach, we used time estimation as a novel and promising marker to monitor negative emotional states^25^. For this, we asked participants to retrospectively rate how fast the period of nausea induction had passed for them. Several studies indicate that acute experimental stress^24^, high levels of anxiety in patients just before they had to undergo chemotherapy^25^, and states of high arousal in everyday life^38^ are associated with a subjective acceleration of time, whilst low-to-moderate arousal levels in everyday life^38^ as well as the unpleasant experience of pain^26^ and waiting^39^ are associated with a subjective “dragging” of time. Our results are consistent with these findings in the sense that acute stress accelerated the passage of time in the untreated groups, just as placebo treatment accelerated the passage of time, but only in the non-stress condition, and presumably by reducing the unpleasantness of nausea. Similar to the findings on gastric myoelectric activity, acute stress may have masked the effects of the placebo intervention on time estimation by inducing effects in the same direction.

This is the first study to investigate the influence of acute stress on subjective and physiological placebo effects in nausea. We applied a randomized controlled, partially blinded design with behavioral, psychophysiological and humoral outcome variables and obtained a high internal validity of the results. Manipulation checks indicated successful induction of acute stress and positive expectations in the respective groups. Nonetheless, the study has several limitations. The relatively homogenous, female-only, young and healthy study population reduces the generalizability of the results. At the same time, variance in physiological placebo effects associated with sex^15,40^ could this way be removed, rendering the results for women more conclusive. Furthermore, even though the overall sample size was considerably large, subgroups were limited in size and small effects could have been missed due to the lack of statistical power. In addition, we focused on nausea induced by a virtual vection drum, which is a well-established experimental model of nausea induction^41^, and we pre-selected participants susceptible to motion sickness. Even though a positive history of motion sickness increases the risk to experience nausea also in clinical settings^42^, the generalizability of our results to other types of nausea, like chemotherapy-induced nausea and postoperative nausea and vomiting, remains to be investigated. Furthermore, the choice of the EGG to measure gastric changes to both placebo and stress interventions can be criticized, as it is knowingly influenced by both, stress and nausea. However, EGG is so far the most promising method for noninvasively measuring gastric correlates of visually-induced nausea^27^ as well as associated placebo effects^15^. Finally, it should be mentioned that the study protocol was retrospectively registered in a public repository. Preregistration has meanwhile been identified as one of the factors associated with increased reproducibility and reliability of empirical studies^43^.

In summary, our results suggest that acute stress did not alter placebo effects on nausea and motion sickness. At the same time, stress appeared to interfere with the validity of the gastric NTT ratio to measure nausea and thus the gastric placebo effect, possibly by increasing normal gastric motility in the fasting state. Future studies should investigate whether placebo effects on the gastric NTT ratio can also be induced in the fed state and how acute stress alters placebo effects in such situations. In addition, the interplay between stress and placebo effects in nausea should also be investigated in healthy men as well as in patients, who are usually exposed to a variety of internal and external stressors.

## Methods

### Study design

We performed a randomized controlled study in healthy female volunteers who were assigned to undergo either a stress or a control task and, after completion, were treated with either a placebo treatment or remained untreated. A small active treatment group (n = 12) was also included to permit blinded administration of placebo treatment (data not analyzed). The study was conducted at the Institute of Medical Psychology, Ludwig Maximilian University (LMU) of Munich in accordance with all relevant ethical guidelines and regulations for human participants. The study protocol was approved by the ethics committee of the Medical Faculty at LMU Munich (# 401–13) and written informed consent was obtained from all participants. The study was registered at the German Clinical Trials Register (DRKS; 08/12/2022, DRKS00027033).

### Participants

Healthy female participants (18–40 years old) were recruited using flyers in Munich universities and postings on social media. We included only women to reduce heterogeneity, as women and men have been found to respond differently to nausea^44^. Further, a placebo effect on nausea-related gastric myoelectrical activity could only be shown in women in our recent study^15^. Interested volunteers were screened for their susceptibility to motion sickness using the Motion Sickness Susceptibility Questionnaire (MSSQ)^45^. If the MSSQ indicated moderate to severe, but not extreme motion sickness susceptibility (score 80–200), participants were further screened for eligibility in a structured telephone interview. Predefined exclusion criteria comprised contraindications for the use of transcutaneous electrical nerve stimulation (TENS; e.g., implanted devices or metal implants, coagulopathies and hypercoagulable states including surgery in the past 4 weeks), history of inner ear disease (e.g., Morbus Ménière, acute hearing loss), acute or chronic somatic and/or psychiatric conditions (i.e. cardiovascular disease, epilepsy, cancer, substance abuse, skin disease), regular use of medications (except for hormonal contraception, L-thyroxine and allergic rhinitis medications), and current pregnancy or breastfeeding. Volunteers with clinically relevant scores on the Hospital Anxiety and Depression Scale (HADS)^46^ were also excluded, using a cut-off point of 8 for anxiety and depression, respectively^47^. Volunteers who met all inclusion criteria and none of the exclusion criteria were invited to a 20-min screening session in the laboratory to test their response to the nauseogenic vection stimulus. Participants who developed at least moderate nausea (5 on a numeric rating scale (NRS) from 0 to 10) for at least 3 min and did not experience extreme nausea (> 9) or vomiting at any point were included in the study.

### Study procedure

To minimise habituation effects to the vection stimulus, the experimental session was scheduled no earlier than 48 h after the screening session. For participants who did not use hormonal contraception, the experiment was conducted during the luteal phase of the menstrual cycle to minimise the effects of gonadal hormones on symptoms, gastric activity, and stress responses^48^. To account for the circadian variability of cortisol levels, all sessions took place between noon and 6 pm^49^. Participants were instructed not to eat or drink anything in the 3 h before the experiment. To ensure the participants’ fasting state, blood glucose levels were determined upon arrival from finger blood samples using a BG Star device (Sanofi-Aventis, Hannover, Germany). Participants were seated in a comfortable chair and asked to complete several questionnaires. The respiration belt and the skin electrodes were attached, physiological measurements were started, and the first salivary cortisol sample was collected.

**Figure 3:**
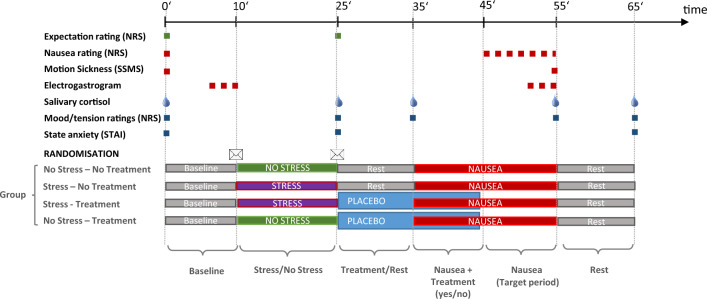
Study design. *Abbreviations: NRS*, Numeric Rating Scale; *SSMS*, Subjective Symptoms of Motion Sickness Questionnaire; *STAI*, State Trait Anxiety Inventory.

The experimental procedure is shown in Fig. 3. After a 10-min resting period during which baseline measurements were taken, participants were randomly allocated to either the stress or the control task, and the respective task was performed. Thereafter, participants were randomly assigned to treatment (active treatment or placebo/sham treatment; only placebo group analysed as above) or no treatment. In treatment groups, the treatment was initiated for 20 min. After the first 10 min of the treatment period, the vection stimulus was applied for 20 min. The experimental session ended with a 10-min resting period. The target period for the statistical analyses comprised the second 10-min period of the vection stimulus, when the treatment had already ended.

### Treatments, randomization and blinding

Participants in the treatment groups received a standardized positive verbal suggestion of nausea improvement (for details, see Aichner, et al. ^14^). Then, a programmable TENS device (Digital EMS/TENS unit SEM 42, Sanitas, Uttenweiler, Germany) was applied. In the placebo treatment groups, the TENS electrodes were attached proximal and distal to a dummy acupuncture point located on the ulnar side of both forearms ^50^, and the superficial massage program of the TENS device was turned on for 20 min to induce a slight tingling sensation at the electrode site^14^. For the real TENS intervention in the active treatment group, the electrodes were placed around ‘PC6’, a validated acupuncture point for the treatment of nausea^51^, and the TENS program was turned on for 20 min. Participants in the control groups did not receive any treatment. They were informed about the importance of untreated groups and were asked at the end of the experiment to rate their degree of disappointment about allocation to the control group. On an NRS from 0 (not at all disappointed) to 10 (extremely disappointed), disappointment was rated low at 2.05 on average (median 1, range 0–7), which makes it unlikely that it affected the results.

The recruited women consecutively received a participant number from a list. Before the start of the study, an independent researcher assigned all participant numbers in advance to the respective experimental conditions using the random sequence generator implemented in Microsoft Excel. For each participant number, the independent researcher prepared two sealed envelopes with respective group assignment information. The first envelope assigned participant numbers to the “stress” or “non-stress” condition and was opened by the person conducting these tasks. After completion of the task, the main experimenter entered the room and opened the second envelope containing the treatment assignment information, thus remaining blinded regarding the stress or non-stress condition. Participants in the treatment groups were blinded regarding the type of treatment (placebo or active treatment), whereas participants in the no treatment control group were necessarily unblinded.

### Nausea induction

Nausea was induced by a vection stimulus generated by a virtual optokinetic drum^13,14^. Vection stimuli create the illusion of self-movement which causes visually-induced motion sickness (VIMS) in susceptible participants^52^. VIMS is frequently used to induce nausea in experimental settings as it avoids pharmacological agents and allows participants to be stationary, which facilitates data acquisition^53^. We induced nausea for 20 min according to an established protocol^13,14^. Black and white stripes moving from left to right with a speed of 60 degrees/second were projected on a semicylindrical semitransparent screen surrounding the participant. The distance between the screen and the participants’ eyes was 30 cm. This way, the projection covered the entire visual field of participants. Participants were advised to look at the screen without fixating on single stripes.

### Stress induction

Stress was induced using the MAST^23^. The MAST combines elements of the cold pressure test^54^ with social evaluation in a mental arithmetic task. Participants in the non-stressed groups underwent a control version of the stress test, involving hand-immersion in warm water and counting instead of performing calculations^23^. Both tests were performed by two persons who were not further involved in the experiment. To participants, the stress test or its control version was described as a “task which may include unpleasant stimuli”.

### Outcome measures and manipulation checks

Nausea intensity was assessed using an 11-point NRS with the poles “not nauseated at all” (0) and “imminently vomiting” (10). To account for the wave-like character of nausea^22^, ratings were obtained every minute during the 20-min nausea induction period and averaged for every 10 min. Motion sickness severity was assessed by the SSMS (adapted from Graybiel, et al. ^55^), with scores of 0 to 3 assigned to responses of none, slight, moderate, and severe symptoms of dizziness, headache, nausea/urge to vomit, tiredness, sweating, and stomach awareness, respectively^14^. The EGG served to examine changes in gastric myoelectrical activity related to nausea (see below). The expected maximum intensity of nausea during the experiment was assessed using an 11-point NRS with poles of “no nausea “ (0) and “imminently vomiting” (10).

Salivary cortisol, mood ratings (11-point NRS from “worst mood” (0) to “best mood ever” (10)^56^), ratings of inner tension (11-point NRS from “no inner tension” to “extreme inner tension”), and state anxiety (State-Trait-Anxiety Inventory; STAI^57^) were used to check the success of the stress task and to examine possible interactions with placebo treatment.

In an exploratory approach, we also assessed time estimation using a 10 cm long visual analogue scale (VAS), asking participants to rate how fast time had passed for them during the 20-min time period of nausea induction. Answers were given by a vertical stroke ranging somewhere between the endpoints of “extremely slowly” (0) to “extremely fast” (10)^39^.

### Physiological measurements

EGG data were recorded with BIOPAC MP 150 device (BIOPAC Systems, Goleta, CA, USA) and AcqKnowledge 4.1 software for data acquisition. We followed the same analysis protocol as in our previous study^15^. In short, after processing the raw data, frequency spectrums during baseline and during the target period were analysed with Fourier transformation and the NTT ratio was calculated as the proportion of mean spectral values of the normogastric frequency band (2.5–3 Hz) and the tachygastric frequency band (3.75–9.75 Hz)^19^. Lower NTT ratios are associated with higher nausea^58,59^. In addition to the EGG, respiratory activity (to control the EGG for respiratory artifacts), and electrocardiogram (results not reported here) were recorded with the BIOPAC MP 150 system.

Salivary cortisol levels were obtained with Salivette Cortisol® swabs (Sarstedt, Germany) at various time points as detailed in Fig. 3. Samples were stored on ice during the session, then centrifuged for 3 min with 3000 rpm at 4 °C and stored at -20 °C. Samples were analysed in duplicate using the IBL International Cortisol Saliva ELISA Kit (catalogue number RE52611) following the manufacturer’s protocol. The sensitivity of the cortisol assay was 0.04 ng/ml and inter- and intra-assay coefficients of variance were < 10%. Individual cortisol values were transformed to logarithmic values with a constant score of 4 added to obtain positive values.

### Statistical analyses

Statistical analyses were performed with SPSS statistics software (version 26, IBM). To address the primary hypothesis testing an interaction of stress and treatment, average nausea ratings during the target period (i.e., during the second half of nausea stimulation; Fig. 3) were subjected to a two-way ANCOVA), with “stress” (yes/no) and “treatment” (placebo treatment/no treatment) as between-subject factors and nausea levels at baseline included as a covariate. Assuming a large effect size partial eta-squared of 0.15 for interaction effects in the analysis of variance, we estimated a priori that 20 subjects per group would be needed to give 95% power to detect a significant difference (with a type 1 error of 5%; calculated by MorePower Version 6.0). Gastric NTT ratio during the second half of nausea stimulation as well as SSMS scores at the end of nausea induction were subjected to two-way ANCOVAs, with the between-subject factors “stress” (yes/no) and “treatment” (placebo/no treatment) and baseline levels included as covariates. Expectation ratings after expectancy manipulation were compared between placebo groups and control groups using a baseline-adjusted two-way ANCOVA with the between-subject factors “stress” (yes/no) and “treatment” (placebo/no treatment). The effect of the MAST on salivary cortisol, mood and inner-tension ratings was evaluated using mixed-design ANOVAs with the within-subject factor “time” (baseline, after the MAST, before nausea stimulation), and the between-subject factor “stress” (yes/no). Greenhouse–Geisser corrected results are reported when Mauchly's test indicated violation of sphericity. Effect sizes are reported as partial eta squared (*η*_p_^2^﻿). Results were considered statistically significant if *p* < 0.05 (two-sided).

## Data Availability

The raw data are available from the corresponding author upon request.
